# Adaptive modulation of microsaccades and saccade dynamics by global luminance

**DOI:** 10.3389/fnsys.2025.1735778

**Published:** 2026-01-14

**Authors:** Chao-Yin Kuo, Chi-Hung Juan

**Affiliations:** 1 Department of Otolaryngology-Head and Neck Surgery, Tri-Service General Hospital, National Defense Medical University, Taipei City, Taiwan; 2 Department of Otolaryngology-Head and Neck Surgery, School of Medicine, College of Medicine, National Defense Medical University, Taipei City, Taiwan; 3 Institute of Cognitive Neuroscience, National Central University, Taoyuan City, Taiwan; 4 Cognitive Intelligence and Precision Healthcare Center, National Central University, Taoyuan City, Taiwan; 5 Department of Psychology, Kaohsiung Medical University, Taoyuan City, Taiwan

**Keywords:** global luminance, microsaccade, pupillometry, saccade metrics, stimulus contrast, superior colliculus

## Abstract

**Background:**

Microsaccades, a type of fixational eye movements occurring during visual fixation, are actively involved in the foveal vision and often linked to various attention and cognitive processes. Moreover, microsaccades are increasingly recognized as part of active adaptive mechanisms to continuously changing sensory environments. However, it remains unclear whether they also adjust to changes in luminance as part of this adaptive mechanism, and whether such luminance-regulated microsaccade responses are functionally significant.

**Methods:**

Total forty participants were recruited in the saccade task with their eye position and pupil size measured by a video-based eye tracker. Participants were instructed to maintain fixation on a central spot and then execute a saccade to a peripheral target stimulus immediately upon detection. We systematically varied the background luminance while keeping foveal luminance constant, by which, allows isolation the effects of global luminance on microsaccade generation. We analyzed the effects of experimental condition (background luminance or stimulus contrast) on microsaccadic responses, microsaccadic suppression effects and the saccadic metrics.

**Results:**

We found that darker background luminance systemically increased microsaccade rates (*F*(2,66) = 4.490, *p* = 0.015) and enhanced saccadic directional accuracy (*F*(2,44) = 8.314, *p* < 0.001). Microsaccades suppressions are significant in all experimental conditions, resulting in reduced saccadic directional accuracy and slower reaction times. Notably, the presence of peri-target microsaccade altered the dynamics of saccades, leading to higher peak velocity, larger amplitude, and greater endpoint deviation.

**Conclusion:**

These findings demonstrate that microsaccade behavior changes as a function of global luminance level, suggesting its adaptive role as part of the oculomotor network. They also suggest a potential role for luminance-driven modulation of superior colliculus activity in oculomotor activities. Taken together, our results offer a new insight into visual–motor coordination under naturalistic conditions.

## Introduction

Luminance changes constantly in natural environment. To adapt the changes in luminance, the pupil constantly adjusts its size to regulate the amount of light reaching the retina. In brightly lit environments, the pupil constricts to facilitate visual acuity by reducing optical aberrations, while allowing sufficient light to maintain a good level of visual sensitivity (Campbell, 1957; Mathot and Ivanov, 2019; Mathot, 2020; Chin et al., 2024). On the other hand, the pupil dilates in dark environments to allow sufficient light for visibility (Mathot and Ivanov, 2019; Wang et al., 2021; Eberhardt et al., 2022). To simulate variable pupil sizes, research utilized a tube with various size of disk (from 1 to 8 mm) as artificial aperture and placed it close to the eye. These studies demonstrated that optimal visual detection and discrimination occur when the artificial aperture size closely matches the natural pupil diameter at a given luminance, highlighting the human eye’s ability to adjust pupil size to optimize visual function across different environments (Campbell and Gregory, 1960; Woodhouse and Campbell, 1975). In other words, pupil size adjusts according to the given luminance level to optimize the trade-off between visual acuity and sensitivity to support visual processing (Mathot and Van der Stigchel, 2015).

Within the framework of active visual adaptation, pupil size may not be the sole effector responding to changes in luminance. Because the SC receives luminance-related sensory input and plays a central role in coordinating oculomotor behavior, it is plausible that eye movement dynamics also adapt to support visual function under varying luminance conditions. Specifically, it remains to be established whether and how changes in luminance drive alterations in microsaccades. Microsaccades, a type of fixational eye movement during visual fixation, also play a crucial role in visual processing (Martinez-Conde et al., 2009). Microsaccades that occur at a rate of 1–2 Hz are saccades with amplitudes within 2 degrees of visual angles, corresponding to the foveal region during visual fixation (Martinez-Conde et al., 2009). Research has shown that microsaccades could counteract neural adaptation and perceptual fading via refreshing the retinal images to maintain visibility during fixation (Martinez-Conde et al., 2004; Martinez-Conde et al., 2006). Moreover, similar to saccadic eye movements that bring objects of interests to the fovea, microsaccades are generated to direct foveal locus to the attended part of objects to provide high-acuity information in the service of ongoing task requirements (Kagan and Hafed, 2013; Poletti, 2023; Kowler, 2024). For example, Ko et al. use a computer-simulated needle-threading task, found that microsaccades are precisely directed toward the location between the thread and the needle (Ko et al., 2010). Furthermore, similar to saccades, the superior colliculus (SC) is causally involved in microsaccade generation (Hafed et al., 2009; Hafed and Krauzlis, 2012). Studies from single-neuron recordings in behaving monkeys have showed that SC neurons discharge in the generation for both saccades and microsaccades, forming a continuity of the spatial representation (Otero-Millan et al., 2008; Hafed et al., 2009; Hafed and Krauzlis, 2012).

In addition to their role in aligning the foveola with object locations of interest, microsaccades are also associated with various visual and cognitive process (Hafed and Clark, 2002; Valsecchi et al., 2007; Siegenthaler et al., 2014; Gao et al., 2015; Dalmaso et al., 2020; Kashihara, 2020; Chen et al., 2021). When a microsaccade is generated around the time of target appearance, target detection thresholds increase, and saccade latencies toward the target are prolonged (Hass and Horwitz, 2011; Chen and Hafed, 2017; Greilich and Hafed, 2024). On the other hand, microsaccade rates and metrics are modulated by both endogenous and exogenous factors (Martinez-Conde et al., 2009). Endogenous factors, such as task preparation, can influence microsaccade behavior (Dalmaso et al., 2020). Studies using the pro- and anti-saccade task have shown lower microsaccade rates during the preparation for anti-saccades compared to pro-saccades (Watanabe et al., 2013; Krasovskaya et al., 2023; Chen et al., 2025). Exogenously, visual stimuli with the negative polarity or smaller sizes often induce higher microsaccade rebound after initial microsaccade rate inhibition (Malevich et al., 2021). A fixation point with smaller sizes compared to larger sizes produces microsaccades with smaller amplitudes and the higher threshold to detect peripheral gabor stimuli (Greilich and Hafed, 2024). Background luminance also modulates microsaccades, with lower microsaccade occurrence correlating with higher background luminance level (Benedetto et al., 2014; Chen et al., 2021). Together, these studies suggest that microsaccade rate and dynamics serve as meaningful indicators of cognitive processes and behavior. While microsaccades play an active role in visual processing and are strongly influenced by high-level processing, it remains to be determined whether they adapt in response to luminance as a mechanism of active vision, and whether this luminance-regulated microsaccade response is functionally significant.

The present study aims to understand how global luminance levels influence microsaccade rate and metrics, and to determine whether and how luminance-driven peri-target microsaccades impact task performance. To investigate this, we systematically varied background luminance levels and target stimulus contrast in a saccade task. A distinction from previous research is that prior studies have investigated the relationship between luminance and microsaccades either in the context of reading-specific tasks (Benedetto et al., 2014), or tasks that do not require subsequent eye movements (Chen et al., 2021). Furthermore, because the SC is causally involved in microsaccade generation (Hafed et al., 2009; Hafed and Krauzlis, 2012) and is modulated by visual contrast (Marino et al., 2012; Marino et al., 2015), changes in background luminance cause changes in visual contrast, and inevitably affect microsaccade behavior (Benedetto et al., 2014; Chen et al., 2021). To avoid this confounding factor, we manipulated global luminance level though altering peripheral background luminance, while keeping foveal luminance constant. We hypothesize that microsaccades, as a mechanism supporting active vision, are modulated by peripheral background luminance through changes in SC activity driven by light input (Gharaei et al., 2020).

## Materials and methods

The current study is based on a new analysis of data from an experiment (Wang et al., 2021), with the task and procedure described in detail previously. Specifically, previous analyses reported the modulation of pupil size on saccadic latency and metrics. Unlike the previously reported analyses from this dataset, here we addressed distinct questions focusing on microsaccadic behavior.

### Experimental setup

All experimental procedures were reviewed and approved by the Institutional Review Board of the Taipei Medical University, Taiwan, and were in accordance with the Declaration of Helsinki (World Medical Association, 2001). Forty participants (mean age: 22.2, SD: 3.6 years, 23 males) were recruited, and participants had normal or corrected-to-normal vision and were naïve regarding the purpose of the experiment. Sample sizes were chosen based on previous studies with comparable microsaccade measurements (Kashihara et al., 2014; Siegenthaler et al., 2014; Dalmaso et al., 2017; Wang et al., 2017; Yep et al., 2018; Dalmaso et al., 2020; Chen et al., 2021). Participants provided informed consent and were compensated financially for their participation.

### Recording and apparatus

Participants were seated in a dark room, with their head stabilized in a chin and forehead rest, and their eye position and pupil size were measured with a video-based eye tracker (Eyelink-1000, SR Research, Osgoode, ON, Canada) at a rate of 500 Hz. Stimuli were viewed binocularly. Stimulus presentation and data acquisition were controlled by Eyelink Experiment Builder and Eyelink software. Stimulus presentation and data acquisition were controlled by Eyelink Experiment Builder and Eyelink software. The stimuli were presented on an LCD monitor at a screen resolution of 1,920 × 1,080 pixels with a refresh rate of 60 Hz, subtending a viewing angle of 43° x 24°, with the distance from the eyes to the monitor set at 70 cm.

### Saccade task

**Figure 1:**
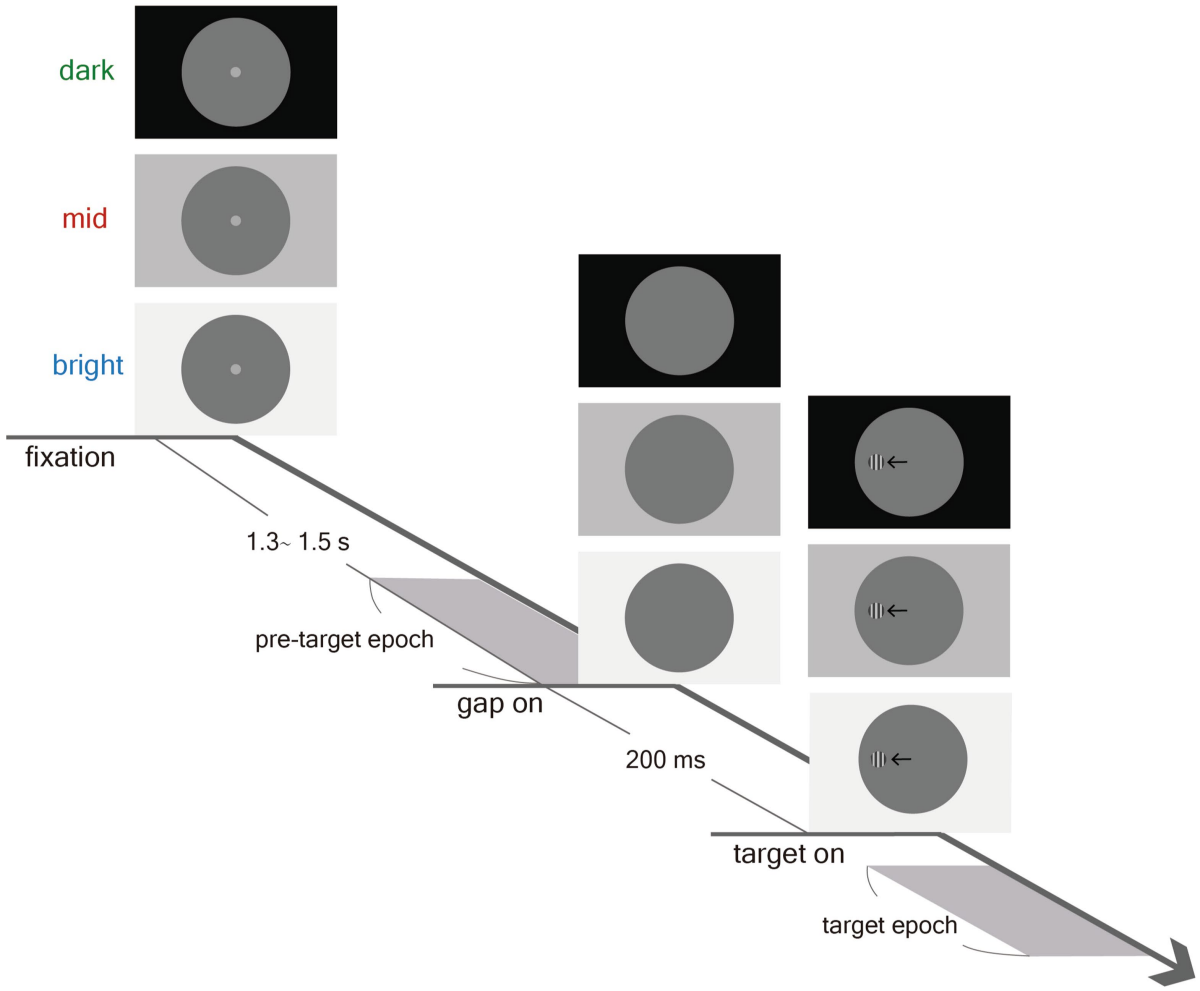
Experimental paradigm. Each trial began with the appearance of a central FP in a large circle (3.5 cd/m^2^) on the background (Bright: 129 cd/m^2^; Mid: 38 cd/m^2^; Dark: 0.1 cd/m^2^). After delay of 1.3–1.5 s, a gap of blank screen was presented for 200 ms before target stimulus presentation, and participants were then required to move their eyes toward the target. Note that the displayed square wave grating circle here is only for illustration of the paradigm.

As shown in Figure 1, participants performed a saccade task in which they were instructed to move their eyes toward a target stimulus immediately upon detection. Each trial began with a gray dot (0.5° diameter, 7.5 cd/m^2^), referred to as the fixation point (FP), overlaid on a large circle (21° diameter, 3.5 cd/m^2^) presented at the center of the screen. Three possible peripheral background luminance levels were used: bright (129 cd/m^2^), mid (38 cd/m^2^), and dark (0.1 cd/m^2^). After 1.3–1.5 s of central fixation, the FP disappeared for 200 ms (gap) before the peripheral target stimulus appeared (0.5° diameter) to the left or right of the FP (5° eccentricity on the horizontal axis). Participants instructed to keep their eyes still during the delay and pre-target epoch, until the peripheral target stimulus was detected. The target stimulus was a circular square wave grating with a clear contour (6 c/deg., two luminance levels), resulting in a stimulus with the same average luminance as the central circular background (3.5 cd/m^2^). Contrast was defined as (Lumhigh − Lumlow)/(Lumhigh + Lumlow), and four levels of stimulus contrast were used: ~12.5%, ~25%, ~50%, and ~100%. The experiment consisted of 586 trials (including 10 practice trials). Target contrast condition (100, 50, 25, 12.5%), peripheral background luminance condition (bright, mid, dark), and target location (left or right) were randomly interleaved. Saccades toward the right and left directions were combined for data analysis.

### Data analysis

**Figure 2:**
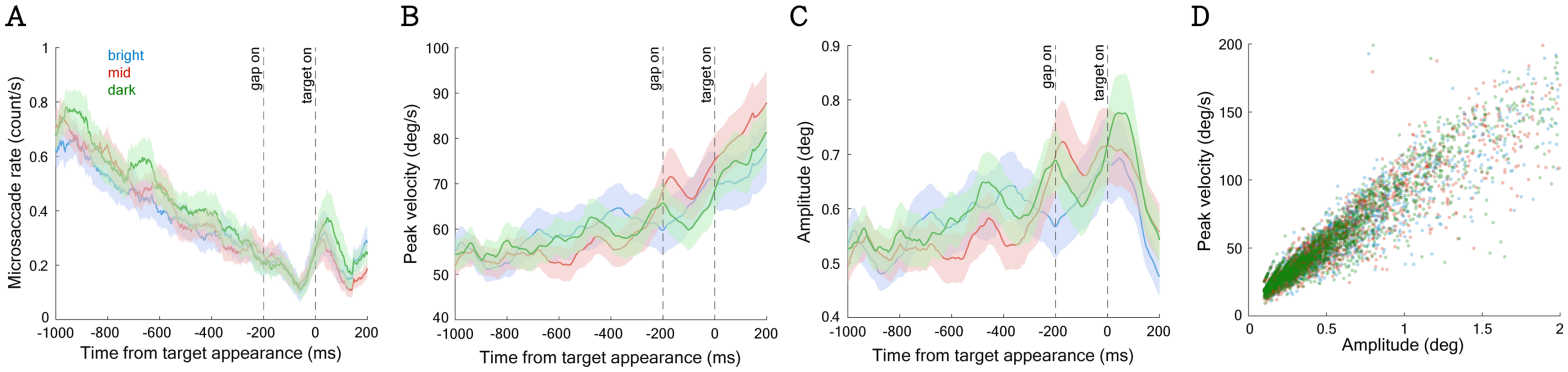
The effect of background luminance on microsaccadic metrics across time and the main sequence slope in pre-target epoch. The overall microsaccade rate **(A)**, peak velocity of microsaccade **(B)**, and amplitude of microsaccade **(C)** across time. The main sequence slope calculated from the pre-target epoch **(D)**.

To detect microsaccades, we followed a well-established method (Engbert and Kliegl, 2003; Engbert and Mergenthaler, 2006), where a velocity-based threshold (threshold: 6 median SDs) was applied on a trial-by-trial basis. In line with previous research (Wang et al., 2020; Chen et al., 2021; Hsu et al., 2021; Wang and Munoz, 2021b; Barquero et al., 2023), microsaccades exceeding the velocity threshold with amplitudes between 0.1° and 2° were included in the analysis. To further reduce noise, we only included microsaccades that occurred simultaneously in both eyes during at least one data sample (2 ms). To investigate the effects of background luminance on microsaccadic responses, we first analyzed an epoch from 800 ms to 200 ms before target appearance (referred to as the pre-target epoch). Each participant had at least 10 microsaccades for analysis, which led to the exclusion of 6 participants from further analysis. As shown in Figure 2D, the main sequence relationship between microsaccadic velocity and amplitude was observed. The amplitude (degrees), peak velocity (deg/s), and main sequence slope (peak velocity/amplitude) of microsaccades were analyzed. Microsaccadic rates were first calculated for each participant (averaged across all trials in each condition) and then averaged across participants for each condition. Similar to previous research (Engbert and Kliegl, 2003; Laubrock et al., 2005; Valsecchi and Turatto, 2009), we calculated the moving average with a moving window of 100 ms to demonstrate the temporal dynamics of microsaccade metrics.

Microsaccadic inhibition and rebound induced by visual stimuli are well- documented (Rolfs, 2009; Hafed, 2011; Martinez-Conde et al., 2013). Since participants were required to move their eyes to the visual target, we could only examine the effects of background luminance and target contrast on microsaccadic inhibition. Given that mean saccade reaction times (SRT) in the highest contrast condition ranged from 166 to 431 ms, the inhibition epoch (referred to as the target epoch) was defined as 50–140 ms after target appearance, to approximately capture the modulation of microsaccadic inhibition before saccade initiation. Prior literature indicates that the microsaccade rate exhibits a temporal signature following target stimulus onset, marked by an initial inhibition period beginning around 50 ms (Reingold and Stampe, 2002), reaching its nadir approximately 100–200 ms post-stimulus, and followed by a subsequent rebound (Engbert and Kliegl, 2003; Rolfs et al., 2008; Malevich et al., 2021). Consequently, the inhibition epoch used in the current experiment covers roughly from the onset to the nadir of this inhibition, and is thus expected to capture the change of microsaccade rate. To investigate the role of microsaccadic responses associated with background luminance levels and stimulus contrast on task-related performance, we also examined microsaccadic suppression effects (Rolfs et al., 2006; Sinn and Engbert, 2011; Watanabe et al., 2013; Hsu et al., 2021). We compared trials with and without microsaccades occurring from 400 ms before to 100 ms after target appearance (Watanabe et al., 2013; Chen et al., 2025), focusing on saccadic performance including saccadic directional accuracy (i.e., correct saccade direction toward the target), SRT, peak velocity, amplitude, and endpoint deviation. Due to generally low microsaccadic occurrence rates, each condition (background luminance or stimulus contrast) retained at least 5 trials for microsaccadic suppression analysis, resulting in 23 and 16 participants being included in the final background luminance and stimulus contrast microsaccadic suppression analyses, respectively. Following previous analyses of saccadic behavior (Wang et al., 2021), SRT was defined as the time from peripheral target appearance to the onset of the first saccade away from fixation, determined by the moment when eye velocity exceeded 30°/s, with an amplitude greater than 3°. Saccade amplitude, saccade peak velocity, and endpoint deviation of the first saccade were also analyzed. Trials were considered correct if the first saccade after target appearance was in the correct direction (i.e., toward the target). Direction errors were identified when the first saccade was away from the target location.

We performed one-way repeated-measure ANOVA to analyze experimental condition (background luminance or stimulus contrast) effects on microsaccadic responses. We also performed 2 × 2 repeated-measure ANOVA (experimental condition and microsaccadic occurrence) to examine microsaccadic suppression effects in each analysis (background luminance or stimulus contrast). Effect sizes (partial eta squared), where appropriate, are also reported. Statistical tests were performed using JASP team (JASP Team, 2019), and MATLAB (The MathWorks Inc., Natick, MA, USA).

## Results

### Background luminance level affected microsaccadic responses

**Figure 3:**
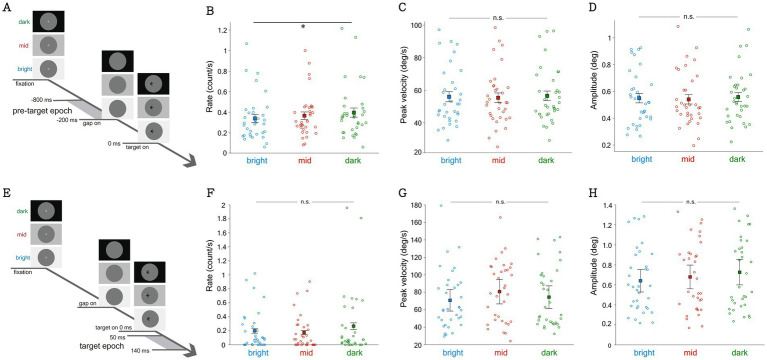
Effect of background luminance on microsaccadic metrics during the pre-target epoch, 800 ms to 200 ms before target stimulus onset **(A)** and target epoch, 50 ms to 140 ms after target stimulus onset **(E)**. Averaged rate **(B)**, peak velocity **(C)**, and amplitude **(D)** of microsaccade during the pre-target epoch. Averaged rate **(F)**, peak velocity **(G)**, and amplitude **(H)** of microsaccade during the target epoch. *Indicates difference is statistically significant (*p* < 0.05).

To investigate whether background luminance level affects microsaccadic responses, we collapsed data from the four contrast conditions and analyzed microsaccadic responses across the three background luminance levels. As shown in Figure 2A, consistent with previous findings (Watanabe et al., 2013; Dalmaso et al., 2020; Chen et al., 2021), microsaccadic rates decreased prior to target appearance, and microsaccadic inhibition occurred approximately 50 ms after task events (e.g., gap or target appearance) (Dalmaso et al., 2017; Wang et al., 2017; Dalmaso et al., 2020; Chen et al., 2021; Hsu et al., 2021; Malevich et al., 2021; Barquero et al., 2023). More interestingly, microsaccadic rates were systematically modulated by background luminance level, with higher microsaccadic rates observed under lower luminance levels during the pre-target epoch (−800 to −200 ms relative to target onset; Figure 3B, F(2,66) = 4.490, *p* = 0.015, ηp^2^ = 0.120). The main sequence slope (the relationship between microsaccadic peak velocity and amplitude) calculated from the pre-target epoch was illustrated in Figure 2D, which demonstrates a similar distribution across different background luminance levels. Microsaccadic peak velocities (Figures 2B, 3C) and amplitudes (Figures 2C, 3D) were not significantly modulated by background luminance levels (*p* > 0.6).

To examine whether microsaccadic responses induced by a saccadic target was modulated by background luminance, we analyzed the epoch for assessing microsaccadic inhibition after target appearance (target epoch: 50 to 140 ms post-target onset) but before saccadic initiation. As shown in Figure 3F, although microsaccadic rates were lower under lower background luminance levels, this trend only approached significance (F(2,66) = 2.418, *p* = 0.097, ηp^2^ = 0.0068). Moreover, microsaccadic peak velocities were not significantly modulated by background luminance (Figure 3G, F(2,66) = 0.898, *p* = 0.412, ηp^2^ = 0.026). Similarly, microsaccadic amplitudes were not affected by background luminance (Figure 3H, F(2,66) = 0.937, *p* = 0.397, ηp^2^ = 0.028).

### Background luminance level affected microsaccadic suppression effects

**Figure 4:**
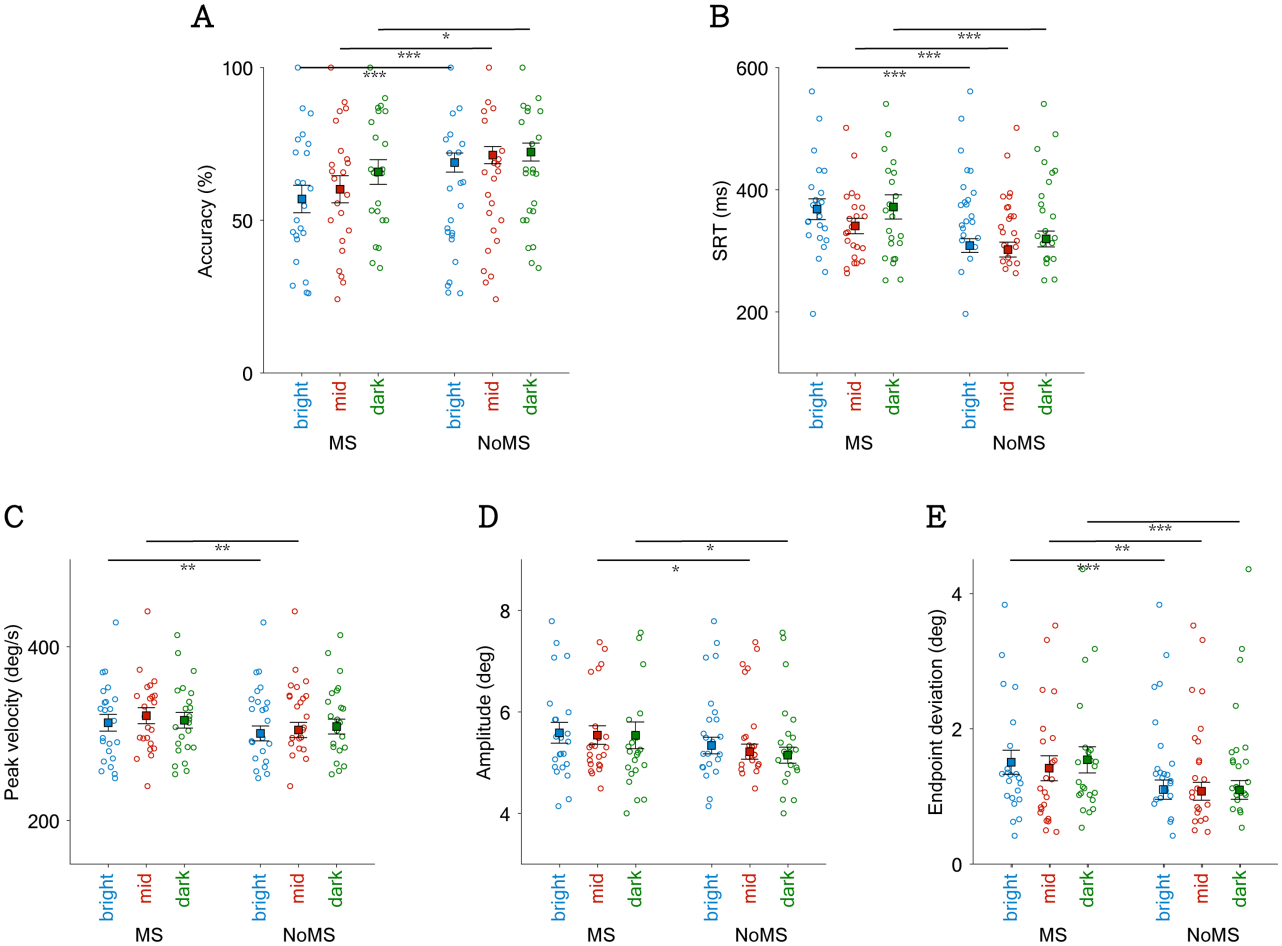
Effect of background luminance and the occurrence of microsaccade on saccadic directional accuracy, SRT, and saccadic metrics. Saccadic directional accuracy **(A)**, SRT of task **(B)**, peak velocity of saccade **(C)**, amplitude of saccade **(D)**, and endpoint deviation **(E)** shown by the presence of microsaccade near target onset with bright, mid, and dark background luminance. *Indicates difference is statistically significant (*p* < 0.05); **indicates difference is statistically significant (*p* < 0.01); ***indicates difference is statistically significant (*p* < 0.001).

**Figure 5:**
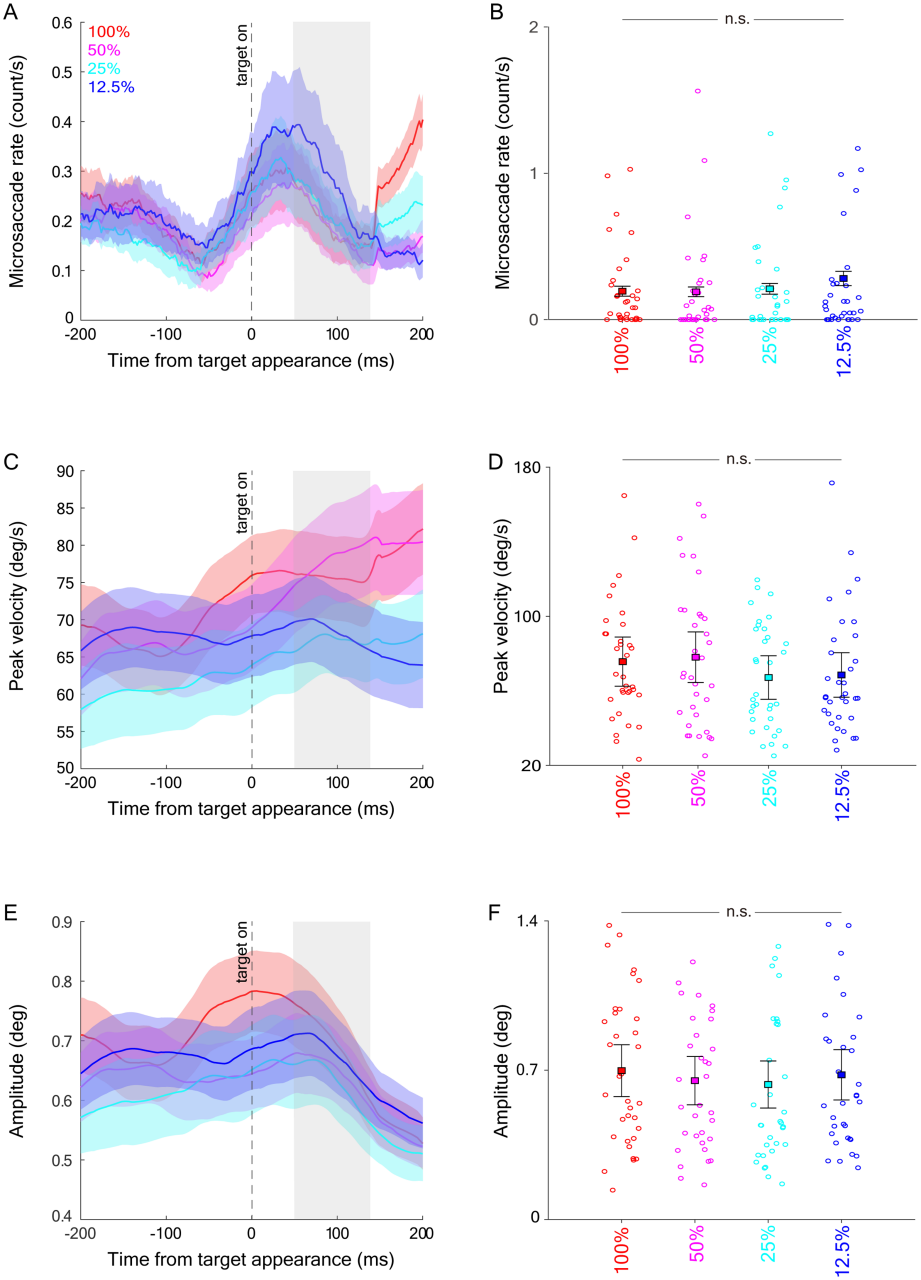
Effect of target contrast on microsaccades during target epoch. Microsaccade rate **(A)**, averaged microsaccade rate during the target epoch **(B)**, peak velocity **(C)**, averaged peak velocity during the target epoch **(D)**, amplitude **(E)**, and averaged amplitude during the target epoch **(F)**. The target epoch is shaded in gray in **(A,C,E)**.

To further investigate the functional significance of microsaccadic responses induced by different background luminance levels, we focused on microsaccadic suppression effects. Specifically, participants performed better when no microsaccades occurred around the time of target appearance (Rolfs et al., 2006; Sinn and Engbert, 2011; Watanabe et al., 2013; Hsu et al., 2021). We separated trials with microsaccades from trials without microsaccades occurring between 400 ms before to 100 ms after target appearance and collapsed data across visual contrast conditions. As shown in Figure 4A, higher accuracies were observed in trials without microsaccades compared to those with microsaccades (microsaccadic main effect: F(1,22) = 17.785, *p* < 0.001, ηp^2^ = 0.447). Background luminance also influenced saccadic directional accuracy, with higher accuracies under lower luminance levels (background main effect: F(2,44) = 8.314, p < 0.001, ηp^2^ = 0.274). However, interaction effects were not significant (F(2,44) = 2.132, *p* = 0.131, ηp^2^ = 0.088). Similarly, significantly faster SRTs were observed in trials without microsaccades (Figure 4B, F(1,22) = 53.371, *p* < 0.001, ηp^2^ = 0.708), and SRTs were modulated by background luminance (F(2,44) = 4.708, *p* = 0.014, ηp^2^ = 0.176), with faster SRTs in the mid-luminance condition. Interaction effects were not significant (F(2,44) = 0.832, *p* = 0.442, ηp^2^ = 0.036). For peak velocity (Figure 4C), significantly lower peak velocities were observed in trials without microsaccades compared to those with microsaccades (microsaccadic main effect: F(1,22) = 14.744, *p* < 0.001, ηp^2^ = 0.401). There was no statistically significant main effect of background luminance on peak velocity (*p* > 0.1), and no significant interaction between background luminance and microsaccade occurrence (*p* > 0.2). Similarly, significantly lower amplitudes were observed in trials without microsaccades compared to trials with microsaccades (Figure 4D, microsaccadic main effect: F(1,22) = 8.827, *p* = 0.007, ηp^2^ = 0.286). There was no statistically significant main effect of background luminance on amplitude (*p* > 0.3), and no significant interaction between background luminance and microsaccade occurrence (*p* > 0.6). Additionally, lower endpoint deviation was observed in trials without microsaccades compared to trials with microsaccades (Figure 5E, microsaccadic main effect: F(1,22) = 23.170, *p* < 0.001, ηp^2^ = 0.513). There was no statistically significant main effect of background luminance on endpoint deviation (*p* > 0.2), and no significant interaction between background luminance and microsaccade occurrence (*p* > 0.5).

### Modulation of visual contrast on microsaccadic inhibition

To examine whether microsaccadic inhibition induced by a saccadic target was modulated by visual contrast, similar to previous analyses, we investigated microsaccadic responses in the target epoch (see Materials and methods). As shown in Figure 5A, microsaccadic rates decreased following target appearance. Contrary to our prediction, microsaccadic rates were not significantly modulated by visual contrast. Although lower microsaccadic rates were observed in higher contrast conditions, these effects did not reach significance (Figure 5B: F(3,99) = 1.678, *p* = 0.196, ηp^2^ = 0.048). Dynamics of microsaccadic peak velocity are shown in Figure 5C, but again, microsaccadic peak velocities during the target epoch were not modulated by stimulus contrast (Figure 5D, F(3,99) = 1.289, *p* = 0.282, ηp^2^ = 0.038). Similarly, microsaccadic amplitudes (Figures 5E,F) were not influenced by stimulus contrast (F(3,99) = 0.350, *p* = 0.789, ηp^2^ = 0.011).

### Visual contrast affected microsaccadic suppression effects

**Figure 6:**
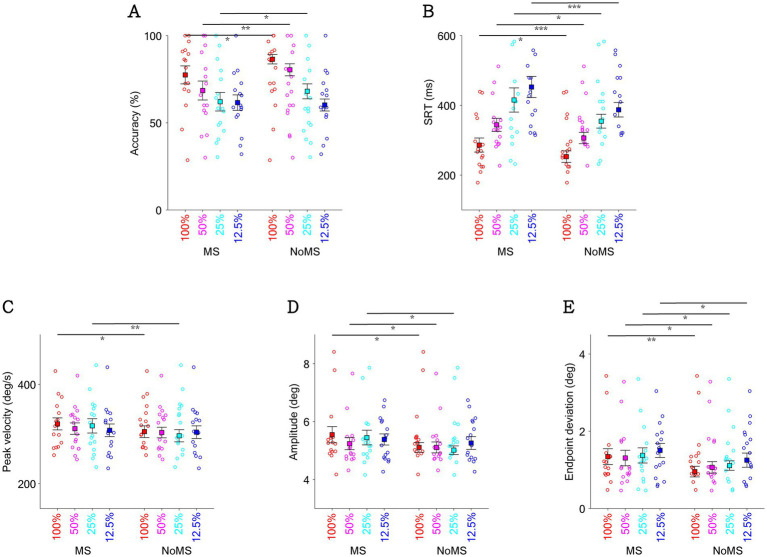
Effect of target contrast and the occurrence of microsaccade on saccadic directional accuracy **(A)**, SRT of task **(B)**, peak velocity of saccade **(C)**, amplitude of saccade **(D)**, and endpoint deviation **(E)** shown by the presence of microsaccade near target onset with different target contrasts. *indicates difference is statistically significant (*p* < 0.05); **indicates difference is statistically significant (*p* < 0.01); ***indicates difference is statistically significant (*p* < 0.001).

To investigate whether visual contrast systematically affected microsaccadic suppression effects, we collapsed trials across the three background luminance levels. Consistent with the preceding paragraph (see also Figure 4), the main effect of microsaccades remains statistically significant for saccade task performance and saccadic metrics (Figure 6). As expected, visual contrast systematically influenced saccadic directional accuracy, with higher accuracies in higher contrast conditions (Figure 6A, contrast main effect: F(3,45) = 23.452, *p* < 0.001, ηp^2^ = 0.610). Interestingly, the interaction effect approached significance (F(3,45) = 2.729, *p* = 0.055, ηp^2^ = 0.154), with particularly pronounced microsaccadic suppression effects in the mid-contrast condition. Visual contrast also systematically influenced SRT (Figure 6B), with significantly faster SRTs in the high-contrast conditions (F(3,45) = 31.470, *p* < 0.001, ηp^2^ = 0.677), while interaction effects were not significant (*p* > 0.4). In terms of peak velocity (Figure 6C) and amplitude (Figure 6D), there was no statistically significant main effect of contrast on either peak velocity or amplitude (*p* > 0.3), and no significant interaction between contrast and microsaccade occurrence (*p* > 0.1). In Figure 6E, visual contrast significantly affected endpoint deviation, with larger endpoint deviations in the low-contrast conditions (contrast main effect: F(3,45) = 6.621, *p* < 0.001, ηp^2^ = 0.306). There was no statistically significant interaction between contrast and microsaccade occurrence (*p* > 0.6).

## Discussion

The present study examined how global luminance modulates pre-target microsaccade behavior and how peri-target microsaccades affect saccadic eye movements during a saccade task. Background luminance level affected task-related performance, with higher saccadic directional accuracy in the lower luminance conditions. Moreover, microsaccade rates were altered by varying luminance, with higher microsaccade rates associated with lower luminance levels. Peri-target microsaccade demonstrate microsaccadic suppression with diminished task-related performance, yielding lower accuracies and slower SRTs. More interestingly, peri-target microsaccades not only affected SRTs, but also modulated dynamics of saccade movements, with higher peak velocities, larger amplitudes, and greater endpoint deviations associated for trials with microsaccades. While the peri-target microsaccade effect cannot be definitively attributed to the background luminance, it suggests an interaction between these oculomotor responses. Together, these findings implicate the coordination of multiple components of oculomotor responses in the service of vision.

### Microsaccades as adaptive responses to global luminance

Successful visual behavior, including fixating on an object, identifying its features, and shifting gaze to new targets, relies on the precise coordination of multiple oculomotor responses. Pupil size adjusts dynamically to optimize visual discrimination and sensitivity under changing luminance (Campbell and Gregory, 1960; Woodhouse and Campbell, 1975; Mathot and Van der Stigchel, 2015; Mathot and Ivanov, 2019); saccades reposition the fovea to capture objects of interest; and microsaccades help maintain foveal image, enhance spatial detail, and support shifts of spatial attention (Engbert and Kliegl, 2003; Martinez-Conde et al., 2004; Martinez-Conde et al., 2006; Donner and Hemila, 2007; Yuval-Greenberg et al., 2014; Lowet et al., 2018; Poletti, 2023). These oculomotor responses are coordinated by the superior colliculus (SC), which integrates sensory, cognitive, and motor signals to support active vision (Hafed et al., 2023). Prior evidence shows that ambient luminance modulates oculomotor activity via the SC for both diurnal and nocturnal species (Delay, 1981). Benedetto et al. (2014) reported that lower screen luminance during reading increased microsaccade rates, reduced saccade velocities, and slowed reading speeds (Benedetto et al., 2014). Similarly, Chen et al. (2021) found that microsaccade rates systematically decrease as luminance increases (Chen et al., 2021). Together, these findings suggest that microsaccade behaviors adapt to luminance variations, likely mediated by the SC. However, previous studies have not fully clarified how global luminance influences microsaccade behavior, because they did not control for the visual contrast of the foveal stimulus. This contrast can modulate fixation-related activity in the SC and, in turn, affect microsaccade behavior (Marino et al., 2012; Marino et al., 2015). Since changes in global luminance are inevitably confounded with changes in foveal stimulus contrast, it is crucial to isolate the effects of global luminance from those of foveal contrast in order to address this question more appropriately.

In the present study, we addressed this gap by holding foveal luminance constant while manipulating peripheral background luminance. This design allowed us to isolate the effects of global luminance on microsaccade generation and examine its relevance for saccadic performance. We found that darker global luminance conditions increased microsaccade rates, suggesting that microsaccades, like pupil adjustments, may serve as adaptive mechanisms that support visual processing, particularly under more demanding low-light conditions (Shlaer, 1937; Rabin, 1994; Otero-Millan et al., 2008). These findings align with evidence that SC microstimulation evokes both saccades and pupil dilation (Wang and Munoz, 2021a), supporting the notion of a shared control mechanism for multiple components of orienting, including microsaccadic and saccadic eye movements as well as pupil responses (Corneil and Munoz, 2014). Furthermore, extensive interlaminar connections within the SC (Hall and Lee, 1997; Lee et al., 1997; Isa et al., 1998; Özen et al., 2000; Doubell et al., 2003), with the superficial layers receiving visual input and the intermediate layers contributing to pupil control, saccadic eye movements, and multisensory integration, suggests that these oculomotor functions are dynamically coordinated to support effective visual performance.

### Role of microsaccade occurrence on task-related performance

Consistent with previous findings (Hafed and Krauzlis, 2010; Hass and Horwitz, 2011; Chen et al., 2015; Chen and Hafed, 2017; Greilich and Hafed, 2024), the occurrence of microsaccades around target onset impaired task performance, as evidenced by reduced saccadic directional accuracy and longer reaction times in trials where microsaccades occurred at target onset. These effects can be explained, at least in part, by microsaccadic suppression, a phenomenon in which microsaccades suppress stimulus-evoked visual bursts in SC neurons by approximately 30% in behaving monkeys (Hafed and Krauzlis, 2010). This suppression begins around 70 ms before microsaccade onset and persists until about 70 ms after microsaccade termination (Hafed and Krauzlis, 2010). Similarly, Chen and Hafed (2017) reported that microsaccades are associated with prolonged SRTs and reduced firing rates in both visual and visuomotor SC neurons (Chen and Hafed, 2017). In line with this, Hass and Horwitz (2011) found that microsaccades not only increase behavioral contrast detection thresholds but also modulate V1 neuronal responses in primates (Hass and Horwitz, 2011). Together, these findings suggest that microsaccade generation suppresses SC and V1 responses to visual stimuli, which likely contributes to the diminished task performance observed in our study. We should also note that the characteristic of a peripheral stimulus could affect the SC neuronal activity and microsaccade effects (Malevich et al., 2022; Wu and Hafed, 2025). Neurophysiological studies have demonstrated how the luminance polarity of a peripheral stimulus leads to perimicrosaccadic changes in the SC, while the microsaccadic suppression was similar for either luminance polarity (Wu and Hafed, 2025).

Extending these findings, we further observed that task performance was more strongly suppressed under bright and mid-luminance conditions. One possible explanation is that larger pupil size under dark luminance facilitates peripheral target detection (Mathot, 2018; Mathot and Ivanov, 2019; Eberhardt et al., 2022), such that even when microsaccades occur, their detrimental effect on performance is mitigated. In contrast, under bright and mid-luminance conditions, peripheral detection performance was already somewhat impaired, even in the absence of microsaccades, likely due to smaller pupil size being less conducive to effective peripheral vision. Without the benefit of a larger pupil, the presence of microsaccades exerts a more pronounced negative impact on task performance (Figure 4A).

Although the impact of microsaccades on saccadic performance has been widely studied (Chen and Hafed, 2017; Dalmaso et al., 2020; Chen et al., 2025), fewer studies have explored their influence on dynamics of saccade movements. We observed a trend of increasing endpoint deviation as target contrast decreased, particularly in the absence of microsaccades (Figure 6E). This finding consistent with previous research showing that stimulus contrast is positively related to SC activity, and inversely related to endpoint deviation (Marino et al., 2012). Notably, saccadic peak velocity has been linked to arousal and mental workload, as it is directly associated with the firing rates of brainstem saccade-generating neurons (Galley, 1989). Previous studies have shown that reductions in peak velocity correlate with fatigue, time-on-task effects, and increased task difficulty, supporting its use as a physiological marker of cognitive state (Galley, 1998; Di Stasi et al., 2010; Di Stasi et al., 2012). Consistent with previous study, we observed a trend of increasing saccadic peak velocity in dark background luminance (Wang et al., 2021), especially when without the occurrence of microsaccade (Figure 4C). The findings that the influence of background luminance and stimulus contrast is more evident when microsaccade are absent, suggesting that the effect of peri-target microsaccade may potentially neutralize the influence of exogeneous factors on saccadic metrics.

Notably, the occurrence of microsaccades around target onset not only affected saccade latencies, but also saccadic metrics, including peak velocity, amplitude, and endpoint deviation, parameters reflecting the dynamics and precision of saccadic control. We found that endpoint deviation increased significantly in the presence of microsaccades, regardless of background luminance or target contrast levels (Figures 4E, 6E). This finding echoes the phenomenon of microsaccadic suppression, as reduced SC activity during microsaccadic suppression may plausibly be associated with less precise oculomotor targeting and larger endpoint deviation. However, saccadic peak velocity (or amplitude) was also increased in the presence of microsaccades. This effect was observed across all background luminance and target contrast conditions, although not all comparisons reached statistical significance (Figures 4C,D, 6C,D). These findings were opposite to our prediction, as reduced target-evoked SC activity due to microsaccade occurrence should result in lower peak velocities, requiring further investigation. Together, these findings suggest that microsaccade may not only reflect aspects of motor preparation but also serve as indicators of underlying cognitive and physiological states.

### Potential neural mechanisms for observed microsaccade effects by luminance level

The SC serves as a central hub in the sensorimotor network, integrating multisensory processing, and visually guided orienting behaviors (Hafed et al., 2023). Its extensive interconnections with cortical, subcortical, and spinal structures enable it to coordinate these functions effectively (White and Munoz, 2011; Martinez-Conde et al., 2013). The SC receives both bottom-up (sensory) and top-down (cognitive or task-directed) input, and sends both ascending projections (to cortex via thalamus) and descending outputs that control the visual grasp reflex via tecto-reticulo-spinal neurons (Corneil and Munoz, 2014). Microsaccade, which are primarily generated by rostral SC, represent key biomarkers of covert orienting and motor preparation within this network (Corneil and Munoz, 2014). Accordingly, microsaccade behavior is modulated by multiple interconnected structures, including the visual cortex, frontal eye field, brainstem reticular circuits (Tse et al., 2010; Martinez-Conde et al., 2013; Corneil and Munoz, 2014; Hafed et al., 2021). More directly, given the SC receives direct retinal signals (White and Munoz, 2011), it is plausible that changes in background luminance influence microsaccade behavior by altering SC activity. In our experiment, changes in global luminance occurred in the peripheral visual field and may have modulated spontaneous activity in the eccentric SC, which has been shown to influence microsaccade dynamics (Buonocore et al., 2021). Background luminance-induced changes in SC activity may serve as a neural mechanism through which ambient lighting conditions modulate the fine-tuning of oculomotor behavior.

Although microsaccadic suppression is a robust phenomenon (Hafed and Krauzlis, 2010; Hass and Horwitz, 2011; Chen and Hafed, 2017), its underlying mechanisms remain incompletely understood. For saccadic suppression, perceptual thresholds increase before, during, and after saccades to maintain perceptual stability (Wurtz, 2008). Thilo et al. generated phosphenes either by transcranial magnetic stimulation at the occipital cortex or by electrical stimulation at the retina and measured the threshold for phosphene perception during saccadic eye movements. They found that the threshold for retina-induced phosphenes increased significantly during saccades, whereas the threshold for cortex-induced phosphenes remained unchanged. These findings suggest that saccadic suppression, reflected by impaired phosphene perception, is mediated early in the visual pathway, likely before cortical processing (Thilo et al., 2004). By analogy, a similar but a smaller-scale context of microsaccade suppression, perception outside the foveal or covertly attended region (Liu et al., 2022, 2023) may also undergo a transient reduction in sensitivity to minimize the effects of image blur caused by microsaccades. However, whether microsaccadic suppression is mediated by the same circuits as saccadic suppression, or whether it additional involves inhibitory effects from the fixational neurons in SC (Munoz and Wurtz, 1993; Everling et al., 1998) or other subcortical and cortical regions, remains to be clarified (Martinez-Conde et al., 2013; Hafed et al., 2021).

Previous studies have shown that both high stimulus contrast and larger pupil size are associated with higher saccadic peak velocity, smaller saccadic amplitude and smaller endpoint deviation (Marino et al., 2012; Wang et al., 2021). In contrast, the presence of microsaccade is linked to higher saccadic peak velocity, larger saccadic amplitude and greater endpoint deviation. This finding suggests that dynamic saccadic metrics are not always tightly coupled and may be modulated through different mechanisms, potentially reflecting distinct physiological functions. Furthermore, the effects of microsaccades may be mediated through neural pathways that are separate from those involved in processing visual stimuli or luminance-modulated pupil dilation. Our observation that the presence of microsaccades attenuates the influence of stimulus contrast and background luminance on saccadic metrics further suggests that microsaccades may operate through a separate neural pathway. A mechanism that may contribute to the relation of microsaccade and saccadic metrics involves the mutual inhibition between saccade-generator and the omnipause neurons (OPNs). The rostral SC drives OPNs and maintains their tonic activity during fixation, while a transient pause in OPNs activity permits saccades and microsaccades generation (Bergeron and Guitton, 2001; Brien et al., 2009; Shinoda et al., 2011). On the other hand, OPNs also modulate saccadic peak velocity: inactivation of OPNs has been shown to reduced saccadic peak velocity while leaving visual fixation intact (Kaneko, 1996; Soetedjo et al., 2002; Miura and Optican, 2006). Furthermore, hypothetical arousal neurons are thought to project onto the OPNs (Gancarz and Grossberg, 1998; Girard and Berthoz, 2005; Rahafrooz et al., 2008), providing a possible explanation for arousal related changes in saccadic metrics (Di Stasi et al., 2013) and suggesting a functional link between microsaccade and changes in saccadic dynamics. Notably, prior work reported that global luminance alone does not modulate saccadic responses (Wang and Munoz, 2021a), implying that the changes in saccadic metrics observed in the present study is likely driven by the presence of microsaccades. However, whether the burst of activity in the rostral SC during microsaccade influence rostral SC that responsible for the tonic activity of OPNs, and thereby affects saccadic metrics remains to be explored.

### Limitations and future directions

In the current study, some trials were excluded due to a low rate of microsaccade occurrence, resulting in fewer than 35 valid trials per condition for analysis. Additionally, the participant pool was limited to healthy young adults, which may constrain the generalizability of the findings. As microsaccade characteristics may vary with age (Port et al., 2016), future research should include a larger number of trials and a more diverse age range. Another essential step is to employ direct neural recordings or high-resolution neuroimaging to examine SC activity under similar luminance manipulation conditions, which would provide critical evidence to support or refine existing models of SC-mediated oculomotor control.

The current task focused solely on peripheral detection under static background luminance. However, luminance in natural environments is dynamic. Future studies should examine microsaccade behavior under temporally varying luminance to better capture natural visual adaptation. Broader task designs are also needed to test how oculomotor responses adjust across perceptual and cognitive demands. For example, the paradigm by Chin et al. may clarify how microsaccades affect discrimination across luminance conditions (Chin et al., 2024); while pro- and anti-saccade tasks under different luminance condition could reveal how cognitive demands modulates microsaccade–saccade dynamics across luminance conditions (Watanabe et al., 2013; Cherng et al., 2020; Krasovskaya et al., 2023).

## Conclusion

The present study highlights the role of microsaccade as adaptive oculomotor responses that adjust to changes in global luminance, suggesting a possible luminance-driven modulation of SC activity. Peri-target microsaccades not only influence perceptual performance during peripheral target detection but also affect the dynamics of subsequent saccadic eye movements, underscoring their relevance in both the dynamics and precision of saccadic control.

## Data Availability

The raw data supporting the conclusions of this article will be made available by the authors, without undue reservation.
